# Taste, Salt Consumption, and Local Explanations around Hypertension in a Rural Population in Northern Peru

**DOI:** 10.3390/nu9070698

**Published:** 2017-07-05

**Authors:** M. Amalia Pesantes, Francisco Diez-Canseco, Antonio Bernabé-Ortiz, Vilarmina Ponce-Lucero, J. Jaime Miranda

**Affiliations:** 1 CRONICAS Center of Excellence in Chronic Diseases, Universidad Peruana Cayetano Heredia, Av. Armendáriz 497, Miraflores, Lima 18, Peru; fdiezcanseco@gmail.com (F.D.-C.); antonio.bernabe@upch.pe (A.B.-O.); vponcelucero@gmail.com (V.P.-L.); jaime.miranda@upch.pe (J.J.M.); 2 Faculty of Epidemiology and Population Health, London School of Hygiene and Tropical Medicine, London WC1E 7HT, UK; 3 School of Medicine, Universidad Peruana Cayetano Heredia, Lima 18, Peru

**Keywords:** hypertension, low-sodium diet, Peru, health knowledge, attitudes and practices, qualitative methods

## Abstract

Interventions to promote behaviors to reduce sodium intake require messages tailored to local understandings of the relationship between what we eat and our health. We studied local explanations about hypertension, the relationship between local diet, salt intake, and health status, and participants’ opinions about changing food habits. This study provided inputs for a social marketing campaign in Peru promoting the use of a salt substitute containing less sodium than regular salt. Qualitative methods (focus groups and in-depth interviews) were utilized with local populations, people with hypertension, and health personnel in six rural villages. Participants were 18–65 years old, 41% men. Participants established a direct relationship between emotions and hypertension, regardless of age, gender, and hypertension status. Those without hypertension established a connection between eating too much/eating fried food and health status but not between salt consumption and hypertension. Participants rejected dietary changes. Economic barriers and high appreciation of local culinary traditions were the main reasons for this. It is the conclusion of this paper that introducing and promoting salt substitutes require creative strategies that need to acknowledge local explanatory disease models such as the strong association between emotional wellbeing and hypertension, give a positive spin to changing food habits, and resist the “common sense” strategy of information provision around the causal connection between salt consumption and hypertension.

## 1. Introduction

Hypertension is an important public health problem especially in low and middle-income countries [1]. In 2006, approximately 14% of the Peruvian population aged 20 and older had hypertension [2]. There is, however, geographical variation of hypertension rates within the country. For instance, in Tumbes, a region located in Peru’s northern coast, one in every four adults aged ≥35 years has hypertension, which is much higher than other regions in the country [3]. 

There are numerous strategies to prevent and control hypertension. One of the more common approaches focuses on reducing sodium intake [4,5], as recommended by several international bodies [6,7,8]. In Latin America, such strategies have translated into regulations to change sodium levels in processed foods, such as bread in Argentina, Chile, and Uruguay [9]. A less common initiative has been to alter the amount of daily sodium intake through the promotion of behavioral changes to reduce the amount of salt consumption at the household level [10]. In Peru, our research team is currently implementing an intervention that aims at reducing blood pressure levels through the distribution of a salt substitute containing less sodium than regular salt, in combination with a social marketing campaign weaved around messages that highlight the importance of reducing the amount of salt used at the household level for everyday cooking [11]. Using a random sample of participants from the six villages enrolled in the study, we found that the average potassium intake in the area is 4.4 (±2.1) g per day (equivalent to about 11 g of salt per day) based on a 24-h urine collection.

Sodium in salt does not only preserve food but also enhances the taste of food [12]. A national study in Peru showed that 20% of those interviewed stated that they add extra salt to food on a regular basis [2]. However, very little is known, on a population-wide basis, about the level of salt consumption recommended as per World Health Organization recommendations of 5 g per day [13]. Therefore, changing sodium intake patterns requires carefully designed interventions. One innovative solution for Peru, where over 80% of subjects in coastal cities have lunch and dinner at home [14] and where daily cooking remains a common practice, is to reduce the amount of sodium in the regular salt by replacing a proportion of sodium with another mineral. 

Peru has a previous successful experience promoting the consumption of iodized salt, whose price is subsidized by the government to ensure its consumption. This sets a positive precedent for the promotion of salt with lower potassium concentrations once we demonstrate the efficacy of such an approach for reducing high blood pressure at the population level.

To tackle a salt-substitution tactic, the first step was to identify the adequate levels of sodium replacement without compromising preferences on flavor that would limit its uptake. To this end, we conducted experiments with sensory discriminatory tests through gradual reduction on sodium concentration [15]. The sodium was then replaced with potassium, which can reduce mean systolic and diastolic blood pressure levels [16,17] and could contribute to the prevention of hypertension, especially in populations with elevated blood pressure [18,19]. We conducted several tests until we identified the combination of sodium and potassium where people were not able to distinguish a dish prepared with regular salt and one prepared with a sodium-reduced salt [15]. 

Once the ideal levels of salt substitution were identified, we had to ensure people would uptake and consume the new product on a sustained manner. Two strategies needed to be put in place: a reliable salt distribution system and a social marketing campaign. Social marketing aims to change a behavior, or introduce a new behavior, by getting acquainted with the target audience and their social environment [20]. In this vein, before embarking on developing such a social marketing campaign, the project team needed general information around local explanations about the causes and consequences of hypertension, the impact of local eating habits, including, but not limited to, salt intake, and its relationship with health problems such as hypertension. Furthermore, it was also relevant to learn the local opinions about the introduction of changes to their regular diet. The data we present was collected during the formative phase of our intervention oriented to promote the use of a salt substitute containing less sodium than regular salt in Tumbes, Peru. 

## 2. Materials and Methods

### 2.1. Study Design

We chose a qualitative approach since our goal was to explore local explanations around specific issues (diet, salt consumption, and hypertension), rather than quantifying or measuring local knowledge on such topics [21].

We used semi-structured interviews and focus groups using a pre-determined set of questions (see Annexes 1, 2, and 3) allowing interviewees to talk about their perceptions around hypertension, diet, salt consumption, and health.

### 2.2. Study Site

The study was conducted in the Tumbes region, in the north of Peru. Tumbes has an approximate population of 200,000 inhabitants, 90% of which live on urban areas [11]. Recent statistics indicate that 3.5% of the population of Tumbes has no formal education and 14.4% of the population was considered poor and 1.4% extremely poor [22]. 

We selected six villages for this study and for the salt substitute intervention. They are all located in rural districts and more than 78% of the population earned less than the minimum wage (750 PEN per month ~240 USD per month). The average population in the villages selected was 600 inhabitants: the smallest village had 507 people and the biggest had 788 people [11]. The main economic activity of one of the six villages was fishing and in the other five, people worked in agriculture. 

While men are mostly agricultural farmers (of rice and plantains), cattle ranchers, or fishermen, women are usually housewives or—in some few cases—owners of small *bodegas*. Women are responsible for cooking at home on a daily basis.

### 2.3. Study Participants and Selection Rationale

We aimed to collect information from the local population differentiated by gender and age. We strove to ensure that participants in the focus group were as homogeneous as possible in order to respond to the need to collect common perceptions and opinions about the research topic, and to help promote a comfortable interaction with and among participants [23]. We wanted to know if there were coincidences or differences in their explanations of the stated research topics. Our goal was to conduct four focus groups per village (24 in total), divided along gender and age groups: one focus group of women from 20 to 44, one focus group of women from 45 to 65, one focus group of men from 20 to 44, and one focus group of men aged between 45 to 65 years old (Table A1). 

Out of the 24 planned focus groups we were able to conduct 14 since it was difficult for people to take time off from their everyday activities. Half of the focus groups were conducted with men. In the fishermen village (Village 3), male participants of the age group 20–44 failed twice to attend and it was decided to replace that focus group with seven individual interviews with men in that age group. In total, 98 participants participated in focus group discussions. 

Besides the focus groups, we conducted interviews with different types of informants: five with health workers from the villages with a primary health care facility (villages: #1, #2, #5, and #6) and twenty with individuals with hypertension. 

People with hypertension were a group of special importance because we thought they would be better informed about causes, consequences, and management of hypertension and could share their experiences about the difficulties of incorporating changes in their diets such as salt intake reduction. Fieldworkers had a hard time finding men with hypertension interested in participating in the study and, as a result, all of our interviewees were female. Interviews with health personnel were relevant because they could provide an overview about the local health perceptions and their health and food habits, based on their regular interactions at the health facility.

### 2.4. Data Collection

#### 2.4.1. Recruitment

Fieldworkers were instructed to go to randomly selected households and ask questions to determine eligibility. If eligible, individuals were invited to participate in the focus group. Fieldworkers faced some difficulties (participants were not at home or declined participation) which affected the randomization process. At the beginning of the focus group session, oral informed consent was obtained. At the end of each session, a short questionnaire containing demographic information (age, sex, and education level) as well as disease status (known hypertension status, years of disease, current treatment if any) was applied. 

Individuals with hypertension were recruited from the baseline data, whose hypertension status was known. Fieldworkers were instructed to look for the randomly selected participants’ households and ask them some questions to determine eligibility. If eligible, they were invited to participate in the in-depth interview. If the participant agreed to participate, a meeting was scheduled. 

#### 2.4.2. Interview and Focus Group Topics

Three different interview guides were developed to collect data about health perceptions, food culture, knowledge of hypertension, and views about salt consumption (whether it is associated with health problems and local perceptions about the amount of salt consumed on a daily basis). There was also a specific question to learn about their expectations around a “new salt” (the salt substitute) which is not part of this analysis. The interview guide for focus groups, individuals with hypertension, and health workers had three common sections: knowledge about hypertension, views about salt consumption, and expectations of a new salt product (Annex 1).

For individuals with hypertension we added questions regarding causes and consequences of hypertension, hypertension management, and experiences with dietary changes for health (Annex 2). For health workers, we added a section about local health needs, local knowledge on hypertension, and their potential role to promote the use of the salt substitute (Annex 3).

### 2.5. Data Analysis

Interviews and focus groups were transcribed and then analyzed using qualitative analysis software (Atlas-ti 7.1). Researchers created a codebook (list of codes and its definition) (Table A2). These codes were used to analyze the contents of the interviews and focus groups. The transcriptions were read to identify information that was relevant for each topic: (1) causes of hypertension; (2) consequences of hypertension; (3) local eating habits and their impact on health, in general and on the onset of hypertension; (4) opinions about introducing changes in the diet. The sections of the interviews or focus groups that had data related to the research topics were highlighted and received a code. With the help of the software, reports were generated for each code. These reports are a word document with a list of quotes (excerpts) of the interviews and focus groups assigned to each code. The reports were read to identify coincidences, repetitions, similarities, or differences in the data [24]. 

### 2.6. Ethics

The study “Launching a Salt Substitute to Reduce Blood Pressure at the Population Level in Peru” was approved by the *Comité Institucional de Ética* (Institutional Review Board) at Universidad Peruana Cayetano Heredia in Lima, Peru on 13 September 2012 (Registration code 58563) and by the Institutional Review Board at Johns Hopkins University in Baltimore, USA on 9 November 2012 (IRB No. 00004391).

## 3. Results

### 3.1. Local Diet, Salt Intake, and Hypertension

#### 3.1.1. Local Diet

Both men and women who participated in focus groups stated that women were the ones responsible for preparing the food at home. Most participants said that they usually eat at home and that they only ate outside the home on weekends or for special occasions. Young men stated they usually eat in “*pensiones*” (*pensión* is the local term for having an economic agreement with a person who will prepare homemade food for you on a regular basis) or restaurants near their workplaces. 

Both in the interviews and focus groups participants mentioned a wide variety of types of meals consumed regularly. Fish was the most mentioned ingredient, and the most common way of preparing it was stewed (*pescado sudado*). The other ingredients consumed on a regular basis were rice, banana, cassava, and sweet potato. Banana (in its different preparations: boiled, fried, or “majado”) was the most consumed carbohydrate. Majado plantain is mashed and then fried with onions and spices. People eat *majado de plátano* both for breakfast and lunch.

*Aliño* was among the condiments women used the most for cooking. *Aliño* is a combination of onion, garlic, achiote, oregano, salt, and oil that is used as a base for most meals. In addition, there is also a frequent use of processed condiments such as *ajinomoto* (monosodium glutamate) and *sibarita* (spice mix), and natural spices such as cumin and pepper. 

“Look, to be honest, I use *aliño* that has onion, garlic, oregano, and a little *achiote*. I grind made it with some oil, a little salt, and I keep it for the week. In my youth, I lived in the countryside (…), I do not like food with artificial colors, I do not like that, I use *aliño*.” (Woman with hypertension, Village 5)

A few women said they did not use condiments for cooking; these women consider condiments to be harmful to health.

According to the health personnel, the local diet is not balanced, since it is based on big amounts of carbohydrates and little intake of vegetables and fruits. This coincides with the information provided by the local people who mentioned the low consumption of vegetables and fruits. Focus group participants explained that such products are out of reach for people in their villages because they are expensive.

Unlike the general population, people with hypertension did mention that they tried to consume vegetables on a regular basis. People with hypertension specified that they followed a special diet, with low consumption of meat and salt.

#### 3.1.2. Hypertension and Salt

Participants associated certain eating habits with health problems. Hypertension was one of such health problems. Women stated that they have heard that salt and spices are related to the onset of some health problems but did not specify which ones. Male participants mentioned that too many spices could cause hypertension but also stressed that overeating, eating too much food or too much fried food, were the culprits of such disease. Young men mentioned that lack of physical activity coupled with eating too much and eating too much salt could be associated with the development of hypertension. 

“…factor number one (for getting hypertension) of course are condiments, and salt… On the first place is the salt, eating a lot of salt makes you prone to developing high blood pressure, and eating in excess, we eat a lot, and [afterwards] what do we do? (We go) to the hammock. That is the problem, (after eating we go) to the bed, to the floor, to the mat, to rest ...” (Man, 18–44 years group, Village 6) 

When asked about the relationship between salt intake and hypertension, only some participants (such as the one in the previous quote) mentioned the excessive intake of salt as a habit associated with the onset of hypertension. Women regardless of their age not only did not understand the process through which salt could cause high blood pressure, but they actually expressed doubts about such a causal relationship. Similarly, male participants explained that they were unclear about the role of salt in relation to illnesses like hypertension. 

“It’s what is said about common salt (that it can cause hypertension), I mean I don’t understand so well how salt influences somebody’s blood pressure.” (Women, 18–44 years group, Village 5)

“But tell me how can it harm you?… I don’t see how it (salt intake) could do harm.” (Men, 45–65 years group, Village 4)

Overall, we found that the interviewed population was confused by the diversity of information about hypertension and about what are “safe” foods for preventing diseases.

### 3.2. Knowledge about Hypertension 

#### 3.2.1. General Information of the Disease

Men and women did not have a clear understanding about the causes of hypertension. They had many doubts about the role of diet in the onset of disease. However, participants did state that hypertension was a dangerous disease because it could kill you. There were no major differences in the general information managed by men and women regardless of their age. 

Unlike the general population, people with hypertension recognized the importance of reducing salt intake to control their blood pressure.

“I eat plain food, I can’t eat salty food because they told me to stop taking salt, [I can] not even add spices, pepper, cumin. They told me not to eat any of that at the [health] center,” (Woman with hypertension, 66 years old).

Usually it has been the health personnel who have explained to them that high salt intake is associated with their elevated blood pressure. 

#### 3.2.2. Causes

According to the general population, the cause of hypertension was related to emotions. Worries, problems, anger, sadness, and strong impressions (getting bad news, for example), were the most frequent explanations for the onset of hypertension. Participants stated that:

“(W)orries can… (cause high blood pressure), over-thinking, worrying can also bring many other illnesses.” (Woman, 45–65 years focus group, Village 5)

“Sometimes (hypertension starts) because of thinking, sometimes because of worries, sometimes because of anger, there are always problems …. (hypertension) comes more from the little outbursts of anger, (or because) you’re thinking about something and then the blood pressure starts to rise or go down, it can be both, but that’s where this disease comes from. It’s not because you eat too much, it’s because of anger, because of the worries that overwhelm you.” (Men focus group, 45–65 years group, Village 2)

Similarly, those with hypertension also argued that one of the main reasons for having hypertension were worries, stress, and *“la pensadera”*, a local term used that refers to over-thinking about a subject that causes concern: 

“It must be that I was thinking about my children. That’s why my blood pressure rises....” (Woman with hypertension, 56 years old)

The emotional origin of hypertension seemed to be reinforced by the health personnel, because some of the participants indicated that the doctors had given them that explanation. 

“The doctor examines me, takes my blood pressure, and tells me that I have emotional hypertension, that I should control it by taking a lot of liquids ....” (Woman with hypertension, 43 years old)

“Because… the doctor explained it to me, she said that this (hypertension) sometimes comes from food, too much salt, or maybe you have troubles at home, or maybe you have something (…), I said I did have a lot of problems in my home, maybe that’s why it happened, too many worries.” (Woman with hypertension, 47 years old)

More than diet, emotional wellbeing was the most common explanation for hypertension in the six villages.

Participants diagnosed with hypertension also acknowledged the role of genetics (e.g., if they had other family members with the disease), being overweight, having a diet based on meat and salt, and having limited physical activity, in the onset and progression of the illness. Some expressed their fear of dying of high blood pressure:

“You know that when you are hypertensive it is like having death between your hands because if I do not take my medication I can die any day.” (Woman with hypertension, 58 years old)

### 3.3. Opinions about the Possibility of Introducing Dietary Changes

Both hypertensive and non-hypertensive participants were asked about their experience and/or their opinion about the possibility of introducing dietary changes to improve their health, such as increasing the intake of fruits and vegetables and reducing the amount of salt they take with their food. 

### 3.4. Salt Intake Reduction

The reduction or elimination of salt from food appears to be a great challenge for those interviewed, both for those diagnosed with hypertension and those who are not: 

“I’ll never give up salt.” (Man, 45–60 group, Village 4)

For some of those interviewed, the idea of stopping their consumption of salt is a saddening one: 

“(W)hen you’re … very sick, they take you to the doctor, right? Then they diagnose you with a bunch of illnesses. Then the diet comes. Because all diseases mean diet. Then they tell you to stop taking salt, and that’s going to be a problem, you get depressed.” (Man, 18–44 group Village 6)

Health personnel state that using a lot of salt on food preparation is part of the local culinary culture and thus very hard to change:

“(T)aking away that [salt] is very difficult, because they are people used to eating everything salty—fish, ceviche, everything. Salt intake is very important here, right?” (Health worker, Village 2)

### 3.5. Fruits and Vegetables

It was found that not only people with hypertension but many other non-hypertensive participants had received advice from the health personnel to improve their diet (i.e., more fruits and vegetables and less fatty foods). Many of these participants, especially those with hypertension, said that it was hard to follow such advice. The difficulties they expressed were associated with the implications of eating less meat and carbohydrates on their strength and their performance on physical tasks. 

“(T)hey told me to eat only carrots ..., only vegetables and nothing else. (They told me) that was my food. (They told me) that I can’t eat anything else. But, who is going to live (like that?)… As I told you, here we work the land, and if you go around with (just) a little bit of carrots… working hard: how are you going to go [to work] like that?” (Man, 45–60 group, Village 3)

Furthermore, as we have mentioned earlier, the economic cost of introducing fruits in their diets was also identified as a barrier for dietary changes, especially by women. Participants were reticent to the health personnel’s suggestion to reduce or stop eating the food they like and enjoy:

“They have already taken away lots of foods from my diet. (It is hard) especially (forme) that I like coffee. They have taken that away from me... the coffee, they have (also) taken away the fish.” (Woman, 45–60 group, Village 2) 

“(Because I have hypertension) they told me I can’t eat chili anymore, but you see I want to eat chili. Even if I die, I want to eat it, it’s so good.” (Woman with hypertension, 56 years old)

“I was diagnosed (with diabetes), like that, and they said “no sugar for 15 days’, and I told the doctor: “I can’t take it, I can’t take it”. They served me my oatmeal without sugar.” (Man, 18–44 group Village 3)

Introducing dietary changes such as increasing the amount of fruits and vegetables and reducing salt intake constitutes a challenge both from the perspective of interviewed population as well as the health personnel that advocate for such changes. 

## 4. Discussion

The goal of this study was to learn about local perceptions about hypertension, diet, and salt consumption that could generate insights for later designing a context-relevant social marketing campaign promoting a salt substitution strategy in rural Tumbes in Peru. 

### 4.1. Main Findings

The results present a context with opportunities and challenges for designing a social marketing campaign aimed at introducing a salt substitute with less sodium content. 

People in the study have some understanding about the connections between certain food habits and health status. Participants said they have heard that eating too much food (in general) or eating too many fatty foods (specifically) could have a negative impact on health. Young men seemed to have a better grasp of this information. However, there was confusion about the way a high-sodium diet impacts health status, since the connection was not clear for them. A study done in three Latin American countries found that people believed that only those who consumed high quantities of salt were at risk of health problems and that only those with high blood pressure or heart problems had to reduce their salt intake [25]. In our study, people with hypertension did mention the importance of having a low-salt diet to manage their disease, but among the general population very few participants stated that having a diet with high salt content could be harmful.

Similar to other studies [26,27], our study found that local people establish a causal connection between emotional problems and the onset of hypertension. This was common between participants with and without hypertension. Worries, troubles, anger, and bad news were identified by both genders and all age groups as the primary cause of hypertension. It is possible that this association is related to the fact that the word for hypertension in Spanish (*hipertensión*) includes the word tension (*tensión*) which stands for “being tense”. A systematic review of qualitative studies around lay perceptions of hypertension (which included 53 studies) found that participants reported stress, food consumption, being overweight, family history, and alcohol as the main causes of hypertension [28]. Participants in the reviewed studies widely and strongly connected stress and worries as a cause, an exacerbating factor, and a consequence of hypertension [28]. We did not find stress being mentioned as a consequence for hypertension, but participants did identify stress as a cause and a worsening factor for hypertension.

Previous experiences of communicational health campaigns suggest that it is important not to assume that because a group is at greater risk of having an illness that they require different communicational strategies [29]. Our results show that there are important similarities in terms of the association between emotions and hypertension among people with and without hypertension. However, besides emotional factors, participants with hypertension in our study mentioned other causes such as salt consumption, genetics, and weight. Weight has been showed to be an important contributing factor of hypertension in the region where this study was conducted [30]. 

Finally, we found among all participants that introducing changes in their diet was seen as something difficult, saddening, and even negative because agricultural work requires physical strength that is associated with a diet rich in carbohydrates. Decreasing the amount of carbohydrates in the diets appears as unacceptable for participants and increasing their consumption of fruits and vegetables is not affordable. Our results suggest that reducing the amount of salt consumed by this particular population is certainly a challenge but one that does not face economic or idiosyncratic barriers.

### 4.2. Implications for the Promotion of a Salt Substitute

Social marketing campaigns for reducing sodium intake have been strongly recommended by the World Health Organization [31]. Social marketing aims at changing individual and social behavior through persuasion [32]. Given that the social marketing campaign of our intervention aims at promoting the use of a salt substitute, we cannot overlook the negative attitude towards dietary changes and the value of taste. Thus, the campaign should frame the reduction of salt consumption in a positive way, such as the improvement in health, and avoid talking specifically about hypertension since people do not make this connection. Furthermore, such a marketing campaign ought to stress the good aspects of the substitute that is being promoted, such as the fact that the taste will be the same. This is particularly important given the role of salt and condiments, such as *aliño* or salad dressings, in the local food culture. Learning about the use of salty “seasonings” in food culture has been shown to be relevant for sodium reduction campaigns in other parts of the world, such as in Ghana [33].

In a US setting, the formative study of the campaign “Skip the Salt, Help the Heart” found that it is not uncommon that people associate a low-sodium diet with bland or tasteless foods [34]. The authors state the importance of using messages that highlight an immediate exchange in benefits, such as that “the taste will be good”, rather than ones that prescribed the importance of consuming less salt on a regular basis, such as: “you will not develop hypertension”. This experience is relevant for our efforts in Peru and shows that despite the geographical differences, there are some relevant lessons when the target population has a high appreciation for salt.

Given that salt is not the only source of sodium, the social marketing campaign could also highlight the importance of learning how to use salty products (such as Monosodium glutamate) moderately as a mechanism for health preservation. One way of helping people to put using less salt into practice could be to introduce a technique for measuring the use of salt during the preparation of meals. Such salt measurements would allow people to identify when they are using too much salt and should consider reducing it. Finally, people should be given alternatives to salt such as other spices available in the local market. 

The connection between emotional health and hypertension should not be overlooked. If possible, it could also be part of a social marketing campaign by stating that just as important as it is to take care of their emotional health it is also important to take care of their physical health. The importance of stress and daily worries management could be articulated as relevant elements for good health, while at the same time emphasizing the importance of healthy eating habits. 

### 4.3. Limitations

One major limitation of our study is that all of the hypertensive individuals interviewed were female and we do not know the perspective of men with hypertension in the selected villages. Some studies show that men use health services less than women and have less knowledge of their health status [35,36,37], however, this cannot be confirmed in our study. Similarly, the study setting and the chosen villages could have flavor preferences and lifestyles that are markedly different from other locations, thus giving a more prominent role to researching local contexts before embarking into behavioral interventions related to the promotion of salt reduction strategies.

Our study was conducted in a rural area where the consumption of processed food with high sodium content such as canned food is rare. Despite this, a lot of sodium seems to be consumed in foods prepared at home such as soup, fried plantains, and *aliño*. In high income settings, most sodium comes from commercial goods and thus if the study was to be replicated in other settings, it should be complemented with questions determining both the total commercial and home cooking sources of sodium in people’s diets.

## 5. Conclusions

This study was nested within the formative phase of an intervention oriented to promote the use of a salt substitute containing less sodium than regular salt. This study clearly showed a number of specific considerations needed to develop a context-relevant social marketing campaign promoting such behavior. First, information about the link between salt intake and hypertension is poor. Yet, proposing a major change in food-related behavior or habit focusing solely on information provision would be inadequate—flavor matters. Second, our findings suggest that a marketing campaign promoting the uptake and acceptance of a salt substitute will not require segmentation of the audience, either by sex or according to hypertensive status. Third, it is relevant to understand local understandings of the disease because despite people with hypertension being more informed about the causes and consequences of hypertension, the primary explanatory model remains the same: emotions are at the root of hypertension. This association is common to findings in other parts of the world, and researchers should make an effort to incorporate it in the strategies for hypertension control.

If the patterns and perceptions around salt intake, wellbeing, and hypertension described in this study are also prevalent in other settings, then these findings can inform similar salt reduction or wellbeing promotion strategies. Most of the paternalistic physician-driven “do not do this” type of messages directly affect the adoption and sustained change towards healthier behaviors, especially if connections between behavior-health-disease are not clear for the target populations, thus limiting the reach and impact of health promotion endeavors. In this sense, conducting preliminary research about the local context and food culture can have a good return for investment in interventions aimed at reducing salt intake, and, importantly, sustaining it over time. As such, either the findings or the approach of this study, or both, can well serve as useful tools in other settings willing to devise similar strategies to stem the tide of hypertension.

## Appendix A

**Table A1:** Number and characteristics of focus group participants per village.

Gender/Age Group	Villages	Number of Participants	Focus Groups
Men ≥45 years old	V2	7	2
	V5	6	
Men <45 years old	V1	6	5
	V2	7	
	V4	7	
	V5	7	
	V6	7	
Women ≥45 years old	V2	6	4
	V3	7	
	V4	8	
	V5	6	
Women <45 years old	V2	10	3
	V4	7	
	V5	7	
**Average**		**7**	**14**
**Total number of participants**		**98**	

**Table A2:** Codes used in the qualitative analysis.

CODE	Definition
Perceptions of High Blood pressure	Perceived gravity of having hypertension.
Symptoms of High Blood pressure	Symptoms associated with hypertension.
Causes of High Blood pressure	Ideas about lifestyles or practices deemed to cause hypertension.
Consecuences of High Blood pressure	Ideas about health aspects that are a consequence of hypertension.
Treatment of High Blood pressure	Practices and information around treating high blood pressure.
Food culture at home	Eating practices at home.
Food culture	Descripción de prácticas relacionadas a la preparación y consumo de alimentos en la comunidad
Knowledge healthy food habits	Information about what are healthy foods.
Practices healthy food habits	Habits and attitudes towards the consumption of healthy food practices
Spice use	Regular use of spices in everyday home cooking
Value of salty taste	Value assigned to the use of salty condiments
Typical local meal	Any reference to the preferred local meal (*plato típico*)
Use of salt for food preparation	Any mention to dishes that require a lot of salt and references to the consumption of such dishes
Salt consumption	Perception of the regular use of salt.
Relationship between salt consumption and HBP	Knowledge and ideas regarding the relationship between salt consumption and high blood pressure.
Person responsible of food preparation	*Decision-maker* of the meals at home.
